# Iterative Image Reconstruction with Under-Sampled Data Assisted by a Neural Network

**DOI:** 10.33425/2771-9014.1012

**Published:** 2023-08-23

**Authors:** Gengsheng L. Zeng

**Affiliations:** Department of Computer Science, Utah Valley University, Orem, Utah, USA; Department of Radiology and Imaging Sciences, University of Utah, Salt Lake City, Utah, USA.

**Keywords:** Image reconstruction, Incomplete data, Under-sampling, Neural Network

## Abstract

**Background:**

Image reconstruction with under-sampled data is usually achieved by an iterative algorithm, which minimizes an objective function. The objective function commonly contains a data fidelity term and one or more Bayesian terms. A popular Bayesian term is the total variation (TV) norm of the image.

**Methods:**

This paper suggests an addition Bayesian term that is generated by a neural network. This neural network is essentially a classifier. This classifier can recognize the artifacts caused by the incomplete data. This classifier is trained by patient images reconstructed by complete and incomplete data sets. This newly introduced Bayesian term is referred to as the CNN score, which is a real number in (−∞, ∞).

**Results:**

Patient studies show the good correlation between the CNN score and the severeness of the artifacts due to the incomplete measurements.

**Conclusions:**

A neural network can extract features from images that are suffering from incomplete measurements and convert the features into a CNN score. An iterative image reconstruction algorithm can be developed to minimize this CNN score to suppress the artifacts in the reconstructed image.

## Introduction

The main motivation for acquiring incomplete data in medical imaging is to reduce radiation dose, as in x-ray CT (Computed Tomography), or to shorten the acquisition time, as in MRI (magnetic resonance imaging). However, image reconstruction using incomplete data may result in poor image quality, with severe artifacts. The traditional analytic image reconstruction methods do not work well when data is under-sampled. Iterative algorithms are commonly used for image reconstruction with incomplete data, similar to other compressed sensing problems. The iterative algorithm is used to optimize an objective function, while the objective function can incorporate some prior information that is not in the measurements.

The objective function for an iterative algorithm typically includes a data fidelity term and one or more Bayesian terms. The data fidelity term encourages the forward projections of the reconstruction to match the measurements, while a popular Bayesian term uses the total variation (TV) norm of the reconstruction [1,2]. Minimizing the TV norm encourages the image to be piecewise constant [3–5].

Machine learning methods have shown promising results in reconstructing medical images with less noise and fewer artifacts. However, the training of a neural network requires a large amount of input/output pairs. In this paper, we assume that good-quality patient images are not available for incomplete measurements. Thus, we cannot use an end-to-end neural network to map incomplete data to its corresponding reconstruction. Despite the lack of good images for the bad measurements, we believe a neural network can still recognize the difference between an image reconstructed with a full dataset and an image reconstructed with an incomplete dataset.

We do not use machine learning methods to directly reconstruct images with incomplete data because we are concerned that the neural network might miss critical details or add spurious details to the reconstructed image [6,7]. Instead, the main goal of this paper is to train a convolutional neural network (CNN) that classifies a ‘good’ reconstruction with a full dataset and a ‘bad’ reconstruction with an incomplete dataset. We then modify the trained CNN to serve as a Bayesian term in an iterative image reconstruction algorithm.

## Methods

When measurements are incomplete, as in few-angle tomography, the imaging problem becomes underdetermined, and naïve filtered backprojection (FBP) reconstruction algorithm produces severe artifacts even when the data is noiseless. These artifacts are sometimes referred to as angular aliasing artifacts [8]. To reduce these artifacts, we need to gather as much prior information as possible to assist the reconstruction algorithm by restricting the solution space. This is why Bayesian terms are important in the objective function for image reconstruction when the measurements are incomplete. The TV norm is helpful in suppressing noise and regularizing the inverse problem, even if the object is not piecewise constant [3].

To further restrict the solution space, if possible, we need additional information. Neural networks may be able to discover more desirable features of a ‘good’ image that differ from the TV norm and to discover more undesirable features of a ‘bad’ image. We propose training a CNN to classify ‘good’ and ‘bad’ images. We believe that obtaining a regression neural network is harder than obtaining a classification neural network. If a neural network is to be used to convert a ‘bad’ image to a ‘good’ image, this is a regression task. Such a neural network requires a large number of bad/good image pairs to train. Each pair must come from the same patient in the same scanning position. On the other hand, to train a classifier, we do not need any same-patient, bad/good image pairs, because the label in a classifier is an integer which represents either ‘good’ or ‘bad.’

The CNN used in our study comprised two convolutional layers, each with 3×3 convolution kernels and a ReLU (rectified linear unit) activation function. The first convolutional layer had 40 channels, while the second had 20 channels. Our images were 128×128 and incomplete projection data consisted of 45 views over 180° with a detector containing 128 detection bins. The imaging geometry was parallel beam.

**Figure 1:**
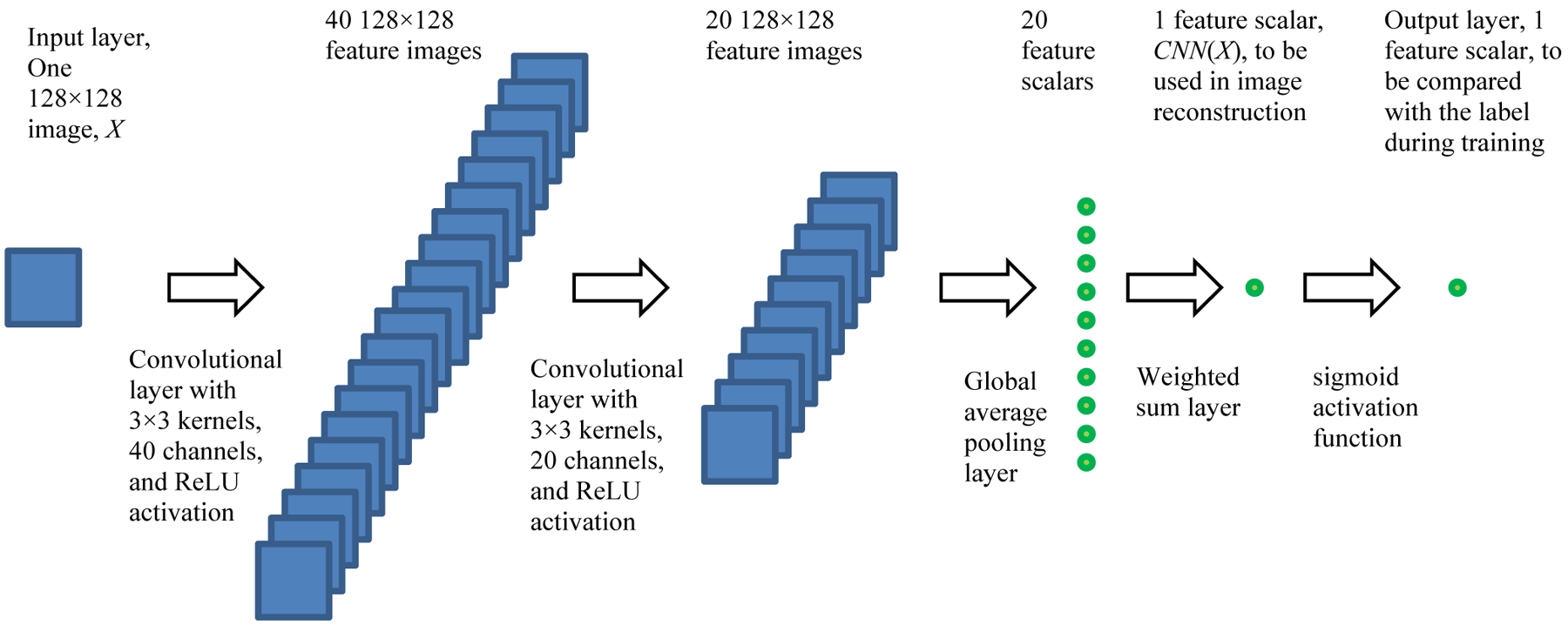
The CNN proposed in this paper to act as a Bayesian term, *CNN*(*X*).

The third layer was a global average pooling layer, which calculated the average value of each channel output as a scalar. These 20 scalars were then flattened into a 20-element vector. The final layer calculated the weighted sum of these 20 elements, and a sigmoid function was used to output the classification results, with a binary output of 1 for a ‘bad image’ and 0 for a ‘good image’. The CNN adopted in this paper is shown in Figure 1.

After training the CNN, the sigmoid function in the output layer was discarded, and the CNN output became a real value in the range of (−∞, ∞). This modified CNN was used as a Bayesian term in the objective function for image reconstruction. The objective function is expressed as:

(1) $$F=\alpha_{1}\|\text{AX}-Y{\|}^{2}+\alpha_{2}\text{CNN}(X)+\alpha_{3}\text{TV}(X)$$

Where *X* is the reconstructed image represented as a vector, *Y* is the measurement vector, *A* is the projection matrix, *TV*(*X*) is the TV-norm of *X*, and *CNN*(*X*) is the modified CNN output value when the input is *X*. In (1), α_1_, α_2_ and α_3_ are three positive parameters set by the user. The objective function *F* can be negative since *CNN*(*X*) may be negative. To optimize the objective function (1), we used an iterative steepest descent algorithm.

## Results and Discussion

The CNN classifier was trained using 4756 patient CT images. Half of them were reconstructed with sinograms of 45 views, which were considered as incomplete datasets with label ‘1.’ The other half were reconstructed with 360 views, which were considered as complete datasets with label ‘0.’ The image size was 128 × 128. The validation split was 10%, and 200 epochs were used. The optimizer was ‘adam,’ and the loss function was ‘binary-crossentropy.’ No bias terms were used in the CNN because they did not improve the classification accuracy.

**Figure 2:**
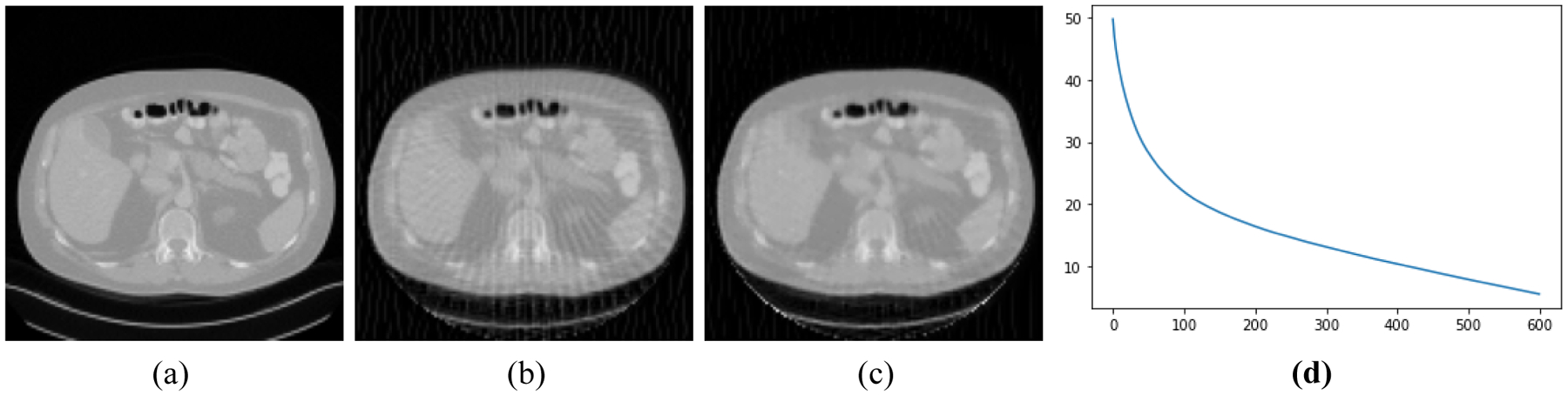
Patient #1. (a) True image. (b) Reconstruction without Bayesian terms. (c) Reconstruction with Bayesian terms. (d) Curve of *CNN*(*X*) vs iterations; the final *CNN*(*X*) is 5.5.

**Figure 3:**
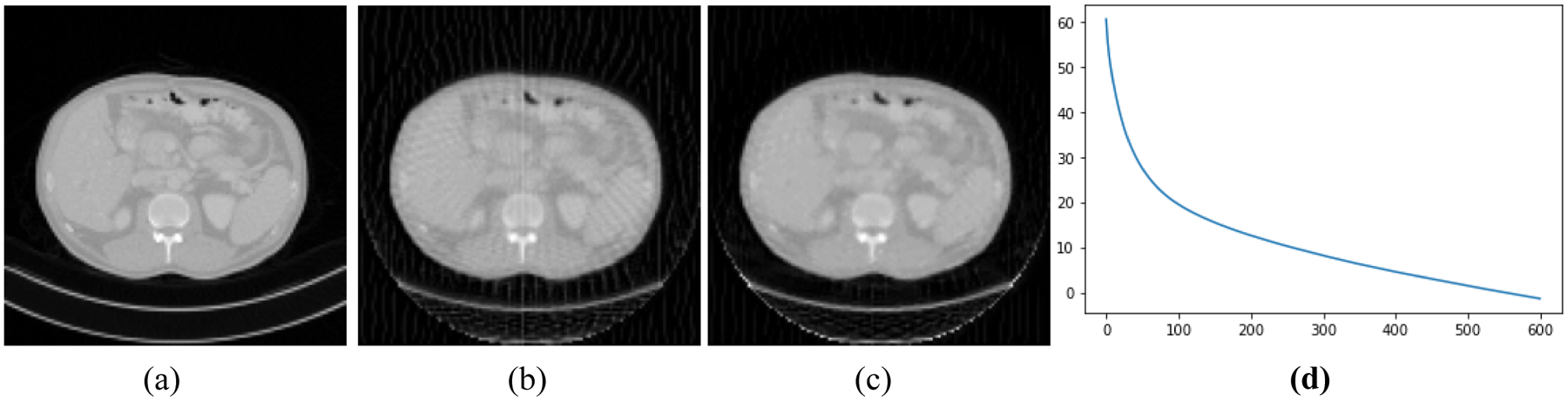
Patient #2. (a) True image. (b) Reconstruction without Bayesian terms. (c) Reconstruction with Bayesian terms. (d) Curve of *CNN*(*X*) vs iterations; the final *CNN*(*X*) is −1.4.

**Figure 4:**
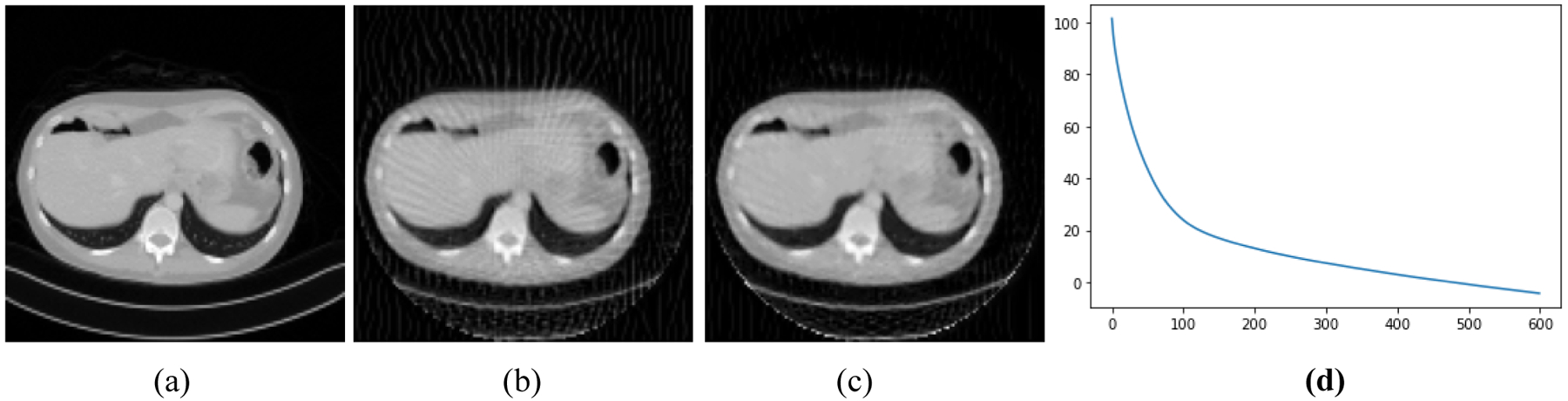
Patient #3. (a) True image. (b) Reconstruction without Bayesian terms. (c) Reconstruction with Bayesian terms. (d) Curve of *CNN*(*X*) vs iterations; the final *CNN*(*X*) is −4.3.

**Figure 5:**
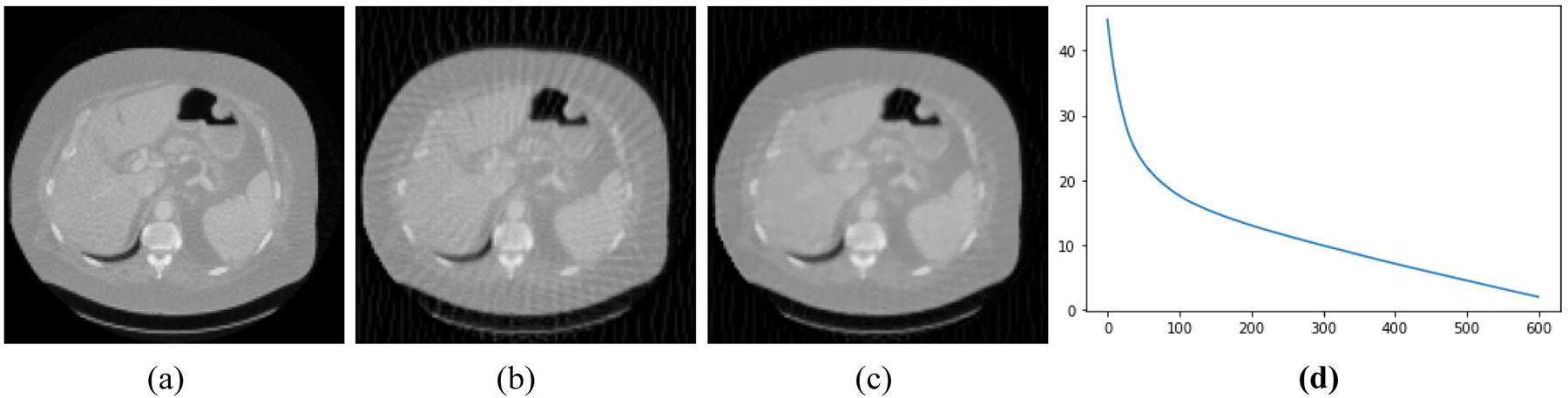
Patient #4. (a) True image. (b) Reconstruction without Bayesian terms. (c) Reconstruction with Bayesian terms. (d) Curve of *CNN*(*X*) vs iterations; the final *CNN*(*X*) is 1.9.

Figures 2–5 present four CT results of iterative image reconstruction with the patients not seen in the CNN training. In these four figures, part (a) shows the gold standard that was obtained by the full data set. In part (b), the angular aliasing artifacts are severe for the images generated by the 45-view data. The results from the proposed method are shown in part (c), where the artifacts are reduced. Part (d) shows the curves of CNN score, *CNN*(*X*), versus the iteration numbers. The larger values of *CNN*(*X*) correlate to severer artifacts; the smaller values of *CNN*(*X*) correlate to less-severe artifacts.

The number of iterations was 600 for all reconstructions. The three parameters were chosen as α_1_ = 0.00000001, α_2_ = 0.002, and α_3_ = 0.00000000001. The artifacts are somewhat reduced by using the Bayesian terms in the objective function. Unfortunately, the artifacts are not completely removed. This unfortunate situation may be caused by many reasons. One cause may be that our neural network model is not adequate to capture the important artifact features resulted from insufficient measurements. Another cause could be that the function *CNN*(*X*) is not convex, and it is difficult to reach the global minimum while in the training epochs. Still another cause might be the theoretical limitation in the sense that when the system *AX* = *Y* is severely underdetermined, the prior information is not strong enough to shrink the solution space small enough to get a useful solution.

**Figure 6:**
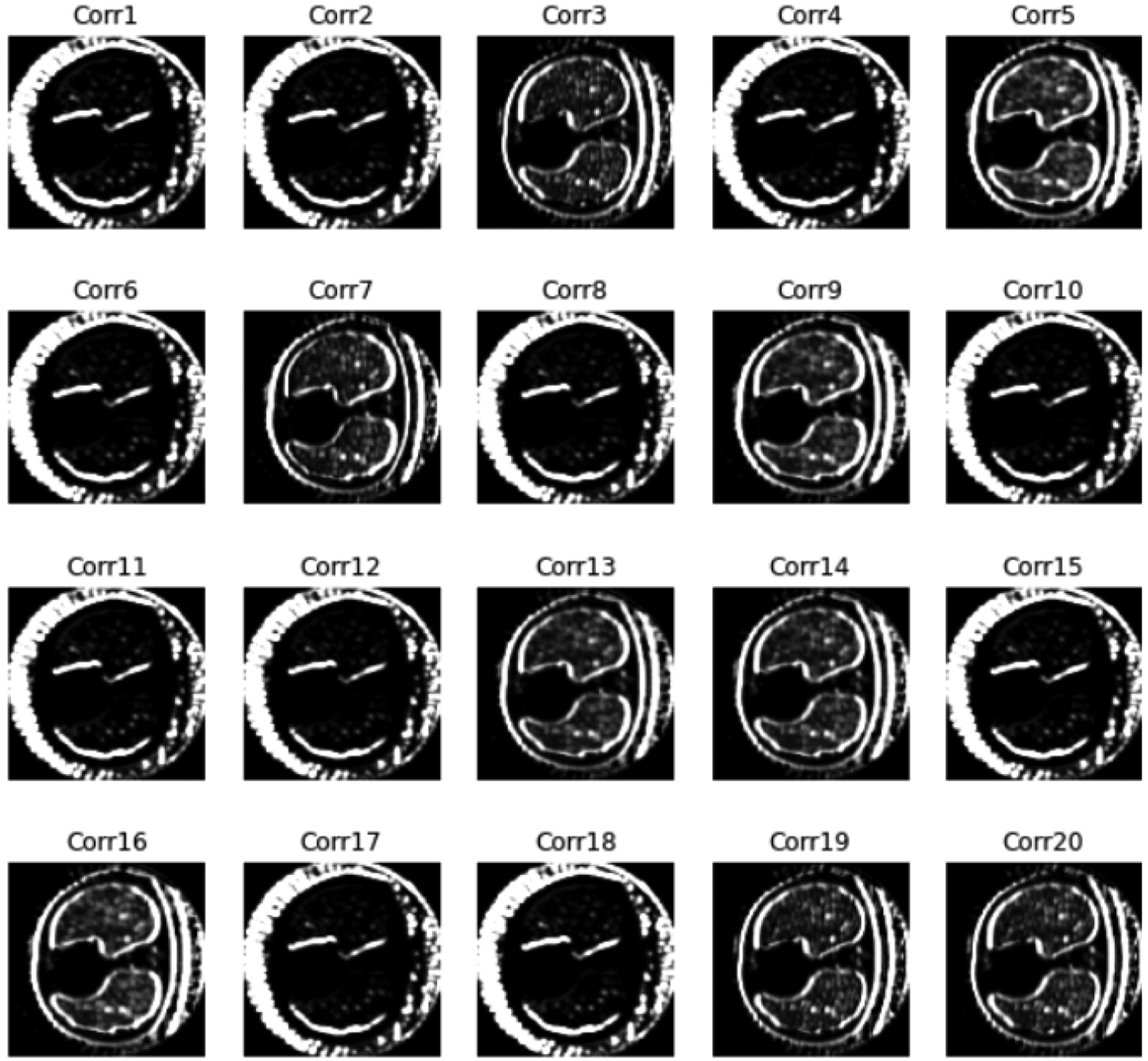
Feature images from the second convolutional layer for an image in the training set using 45 views.

In Figure 6, we display 20 feature images from the output of the second convolutional layer. The input image was obtained by 45 projection views and contained severe artifacts. It seems that the CNN is an edge detector that extracts the object boundaries. The edge detector can also catch the streaking artifacts. If the image has many streaking artifacts, the CNN score, *CNN*(*X*), tends to have a large value. This observation may explain how the CNN score can be used to assist image reconstruction.

## Conclusion

When measurements are under-sampled, the system *AX* = *Y* is under-determined. In this case, prior information about the image becomes important to restrict the solution space, so that the solutions in the shrunk solution space are useful in practice.

The TV norm has been shown to be helpful. It would be nice, in addition to the TV norm, if we can find some other prior information about the image. We are hoping that a neural network can capture more features of the artifacts than the TV norm can. This paper suggests that a neural network classifier be used to catch some features of the artifacts, because neural networks, in general, are excellent at extracting features from a class of objects and can be trained to recognize artifacts. The main goal of this paper is to use a neural network recognizer as a Bayesian term in an objective function for iterative image reconstruction with under-sampled data. The feasibility of this approach has been demonstrated in some patient CT studies.
